# News, views and where best to choose

**DOI:** 10.1113/EP092477

**Published:** 2025-01-01

**Authors:** Ken D. O'Halloran

**Affiliations:** ^1^ Department of Physiology University College Cork Cork Ireland

After serving three consecutive terms as Senior Editor of *Experimental Physiology* (2015–2024), I am hanging up my boots. Or perhaps that's better put as—retiring my quill! What privilege and pleasure the opportunity provided me. Truly!

I had the great advantage of working with successive Editors‐in‐Chief (Paul McLoughlin, Mike Tipton and Damian Bailey), each of whom strengthened the journal through their respective initiatives and steady leadership, leveraging the boundless energy of the Editorial Board and staff of The Physiological Society. Advantage too, in handling a steady flow of high‐quality studies illustrating the majesty of nature viewed through the lens of our beloved discipline.

As an Editorial Board, we wrestled with a rigour and reproducibility crisis and the challenges associated with a complete transition to open access (Bailey & Stewart, 2022), amongst other matters that created headache. We traded on tradition but needed to evolve and innovate to survive. Change is unsettling, and we tested the temperament of our community. Some of our decisions were hand crafted, others handed down, fashioned by external forces. Part watchmaker's forceps, part sledgehammer! As a Board, we may not have always agreed, but we were always agreeable. Always respectful. Oh, how that basic tenet of debate has changed in the wider world during the arc of my tenure! Rest assured it remains a staple behaviour at *Experimental Physiology*.

Although there are many aspects of the role that I relished (and continue to do so over on the other channel at *The Journal of Physiology*), three distinct aspects reveal themselves most clearly. Frankly, they each warm the cockles of my heart.

During my tenure, I have strived for thorough, fair‐spirited, value‐added peer review to help authors and the journal present the best version of the science (Berg et al., 2024). *Experimental Physiology* is deeply indebted to a cadre of referees, including but extending well beyond the reviewing editors on the books. Collectively, they consistently provide high‐quality constructive critique of submitted articles. This facilitates the job of a Senior Editor greatly but offers challenges too, as it should, when opposing views present, as they frequently do. Such moments of tension often teach us the most, if we have the patience to pick them apart. I have benefitted enormously from the collective wisdom of the community of researchers engaged in this curious practice of ours. I have sought to add value where I could, always anchored to the responsibilities I bore on behalf of the authors, referees, the journal and the respective fields of study.

Success of our authors should not go unmarked. I had the privilege of contributing 41 viewpoints on a wide range of research topics in cardiorespiratory physiology, providing perspectives on the value of interesting papers and taking the opportunity to widen my own vista with further reading. Many of these adventures were shared enterprise with laboratory members, helping them to learn the craft (or learning from them!). The collective ambition under the current leadership of the Journal for more editorial content, including invited editorials across a wide range of contemporary important issues, is laudable. The need for informed, balanced, evidence‐based commentary from trusted sources has never been greater or more important for the wider readership (Drummond & Tipton, 2024).

I have greatly enjoyed the many interactions with the community of editors, reviewers and authors, along with the staff of The Physiological Society, especially Diana Jones throughout my time at *Experimental Physiology* and Alex Stewart in more recent years. I continue to enjoy their company and benefit from their professionalism.

In closing, a look to the future. I share the view espoused by the current Editor‐in‐Chief, Damian Bailey, that Editors should demonstrably support the journals they serve through provision of original research articles. As in other walks of life, we should lead by example. I have and will continue to submit original research articles and review articles to *Experimental Physiology*. But of course, we have choice and other obligations. A good chunk of my content is directed to our sister journal, *The Journal of Physiology*, where I serve as Senior Editor. And occasionally I stray elsewhere, sometimes for no good reason, which I have recently contemplated and will rein in! It is critical that as a community we support Society journals. I am motivated to do so, in part owing to a sense of loyalty in recognition of long‐standing tradition. However, I am especially motivated to do so because of the quality of Society journals and the broad benefits that charitable societies bestow on members and society at large. *Experimental Physiology* is a quality journal, a home for quality science. I encourage you to submit your next article here. I will be sending mine. We can trust that our work will be in safe hands!

## AUTHOR CONTRIBUTIONS

Sole author.

## CONFLICT OF INTEREST

The author declares no conflict of interest.

## FUNDING INFORMATION

None.
